# Supplementary figures and images for: Combining molecular dynamics simulations with small-angle X-ray and neutron scattering data to study multi-domain proteins in solution
Source: PLoS Comput Biol. 2020 Apr 27;16(4):e1007870. doi: 10.1371/journal.pcbi.1007870 (PMC7205321; doi:10.1371/journal.pcbi.1007870)

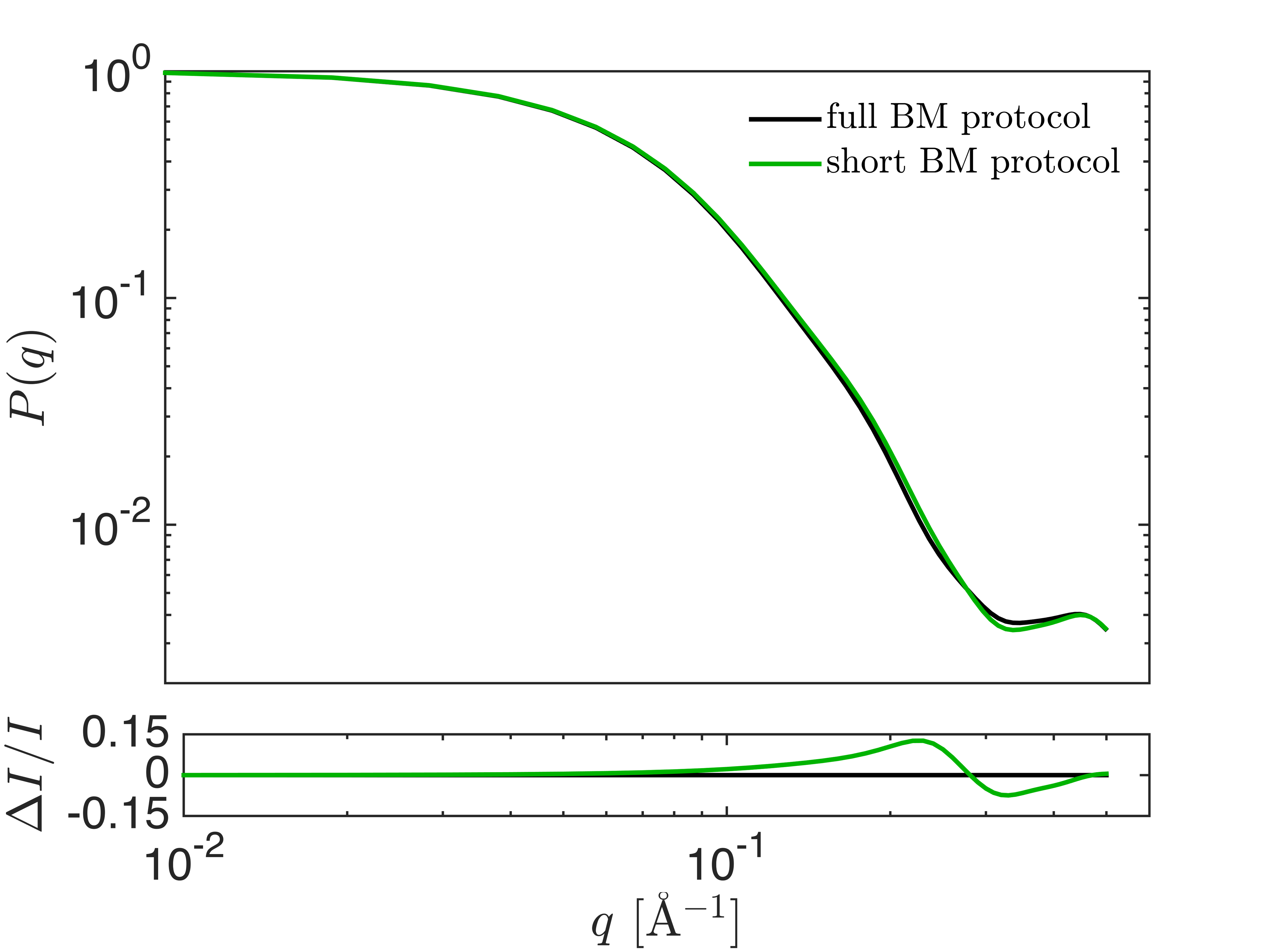

Supplement: S1 Fig — Calculated theoretical form factor P(q) = I(q)/I(0) for a representative frame after the full back-mapping protocol (black line) and a shortened back-mapping protocol (green). See Methods section for more details. Residuals show the relative difference. (TIF) [file pcbi.1007870.s001.tif]

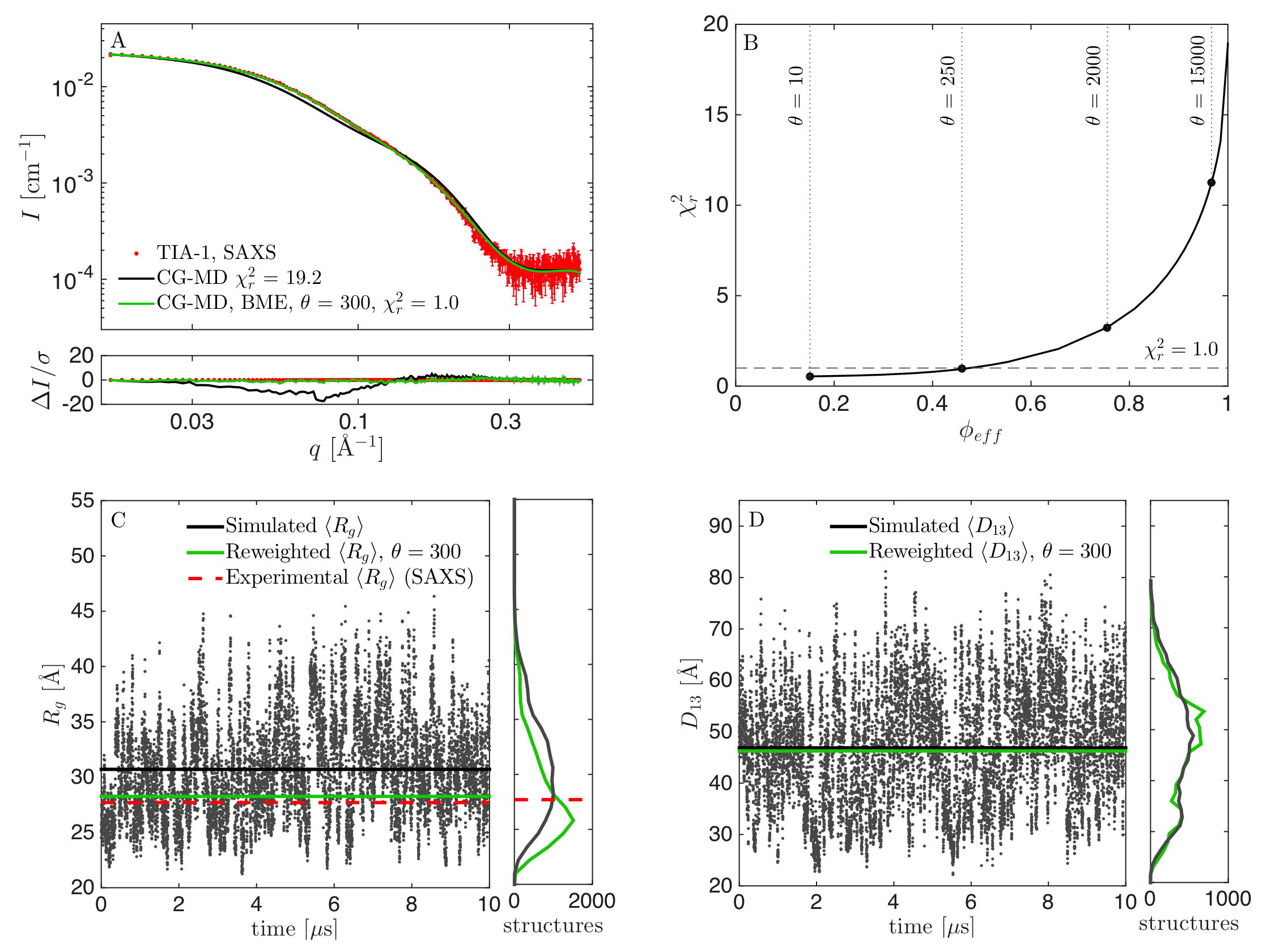

Supplement: S2 Fig — (A) Fit to SAXS data with adjusted force field before (black) and after reweighting at θ = 300 (green). (B) χr2 vs. ϕeff for selection of θ. (C) Rg calculated from structures during the simulation (black), experimental Rg from SAXS (red), and mean Rg from the reweighted ensemble (green), with corresponding histograms in the right panel. (D) Calculated D13 before and after reweighting. (TIF) [file pcbi.1007870.s002.tif]

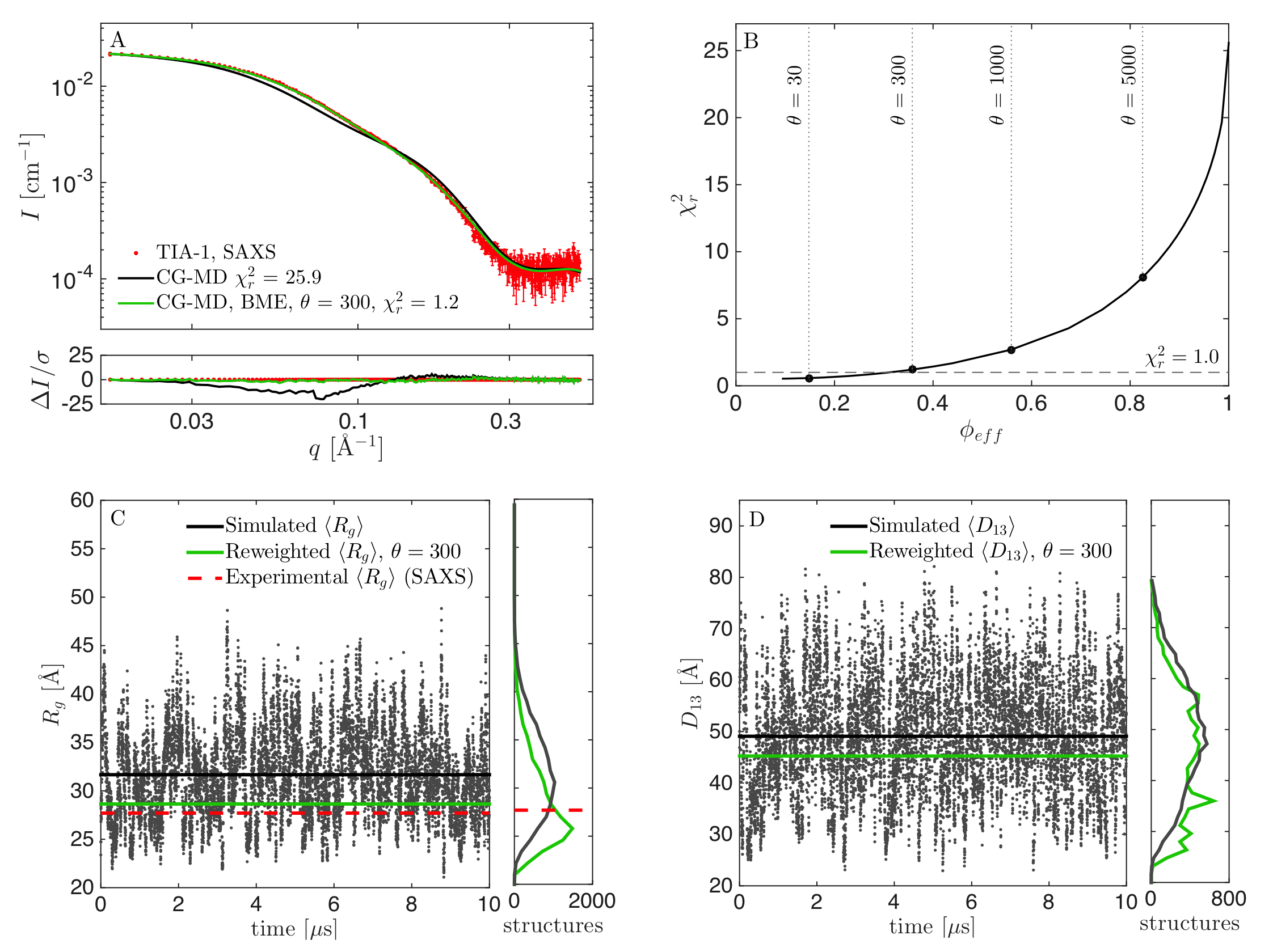

Supplement: S3 Fig — (A) Fit to SAXS data with adjusted force field before (black) and after reweighting at θ = 300 (green). (B) χr2 vs. ϕeff for selection of θ. (C) Rg calculated from structures during the simulation (black), experimental Rg from SAXS (red), and mean Rg from the reweighted ensemble (green), with corresponding histograms in the right panel. (D) Calculated D13 before and after reweighting. (TIF) [file pcbi.1007870.s003.tif]

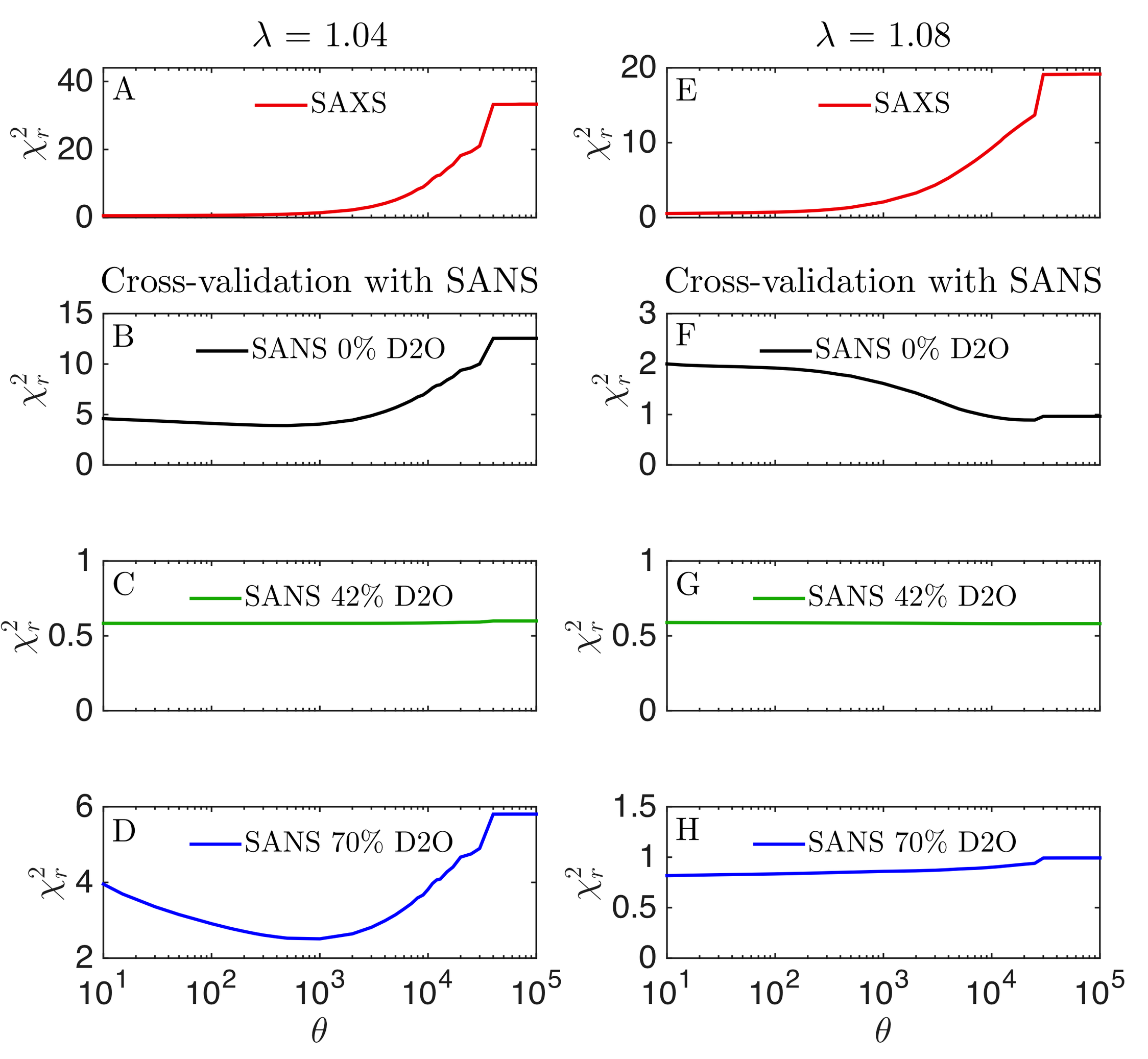

Supplement: S4 Fig — Results of SAXS reweighting (A, E; red) cross-validated with SANS at 0% D2O (B, F; black), SANS at 42% D2O (C, G; green), SANS at 70% D2O (D, H; blue). Simulated at (A, B, C, D) λ = 1.04, and (E, F, G, H) λ = 1.08. (TIF) [file pcbi.1007870.s004.tif]
