# Supplementary material for: Combining molecular dynamics simulations with small-angle X-ray and neutron scattering data to study multi-domain proteins in solution
Source: PLoS Comput Biol. 2020 Apr 27;16(4):e1007870. doi: 10.1371/journal.pcbi.1007870 (PMC7205321; doi:10.1371/journal.pcbi.1007870)
Supplement: Attachment — Submitted filename: __tia1__rebuttal__5__lindorff__2020__.pdf [file pcbi.1007870.s005.pdf]

UNIVERSITY OF COPENHAGEN
FACULTY OF SCIENCE

Peter M Kasson. Associate Editor
PLOS Computational Biology

Revision of manuscript (PCOMPBIOL-D-19-02231)

15 MAR 2020

Thank you very much for sending us the reviewer comments on our paper "***Combining molecular dynamics simulations with small-angle X-ray and neutron scattering data to study multi-domain proteins in solution***". We were very pleased with the very positive response and the useful comments and questions. We have now addressed those in a revised version of the manuscript, which we attach. A point-by-point response to the comments/questions follows this email.

Yours sincerely,

Prof. Kresten Lindorff-Larsen

PROFESSOR OF COMPUTATIONAL PROTEIN BIOPHYSICS

DIRECTOR OF THE NOVO NORDISK FOUNDATION CENTRE
PRISM: PROTEIN INTERACTIONS AND STABILITY IN MEDICINE AND GENOMICS

DIRECTOR OF THE LUNDBECK FOUNDATION BRAINSTRUC INITIATIVE IN STRUCTURAL BIOLOGY

MEMBER OF THE LINDERSTRØM-LANG CENTRE FOR PROTEIN SCIENCE &
THE STRUCTURAL BIOLOGY AND NMR LABORATORY

DEPARTMENT OF BIOLOGY,
UNIVERSITY OF COPENHAGEN

OLE MAALØES VEJ 5
2200 COPENHAGEN N

lindorff@bio.ku.dk
www.bio.ku.dk/sbinlab
+45 4267 0074

Reviewer #1:

Larsen et al. present an insightful application of their Bayesian method for (minimal) ensemble reweighting. The study is well designed, the conclusions are justified by the data, and the manuscript is exceptionally clearly written. I strongly appreciate the extensive discussion on the strengths and limitations of the reweighting protocol, which will guide future efforts. Plos Comput Biol is certainly a suitable journal for this work.

I suggest only a few clarifications. Further review is not needed. Congratulations to this insightful work!

We thank the reviewer for his reading and comments on our paper, and for the questions below. We are also very glad to hear that the reviewer liked our work and the writing.

1) page 4:

"Resolution effects were included in the Pepsi-SANS calculations, using the uncertainty of the measured -values, as provided by in the fourth column of the SANS data."

How exactly is this done? The Pepsi-SANS documentation does not provide much information.

We apologize, and now provide more details on how this is done. Briefly, we apply a Gaussian convolution to the theoretical I(q) curve with a Gaussian of the form, $\exp(-x^2/(2\,\sigma^2))$, where σ is the standard deviation taken from the forth column of data. (p. 4; Page numbers refer to the version with tracked changes.)

2) phi_eff gives the fraction of frames that dominate the final, reweighted ensemble. I was wondering if you can translate phi_eff into a the error of the force field (in terms of free energies). Does a certain phi_eff (e.g. 1%) mean that the force field is wrong by XX kilojoule per mole? Such translation would give phi_eff a more intuitive meaning.

This is a very good question and one we have ourselves discussed extensively. The two are certainly related because the KL divergence is related to the free energy of changing one energy surface into another (Qian, 2001, https://doi.org/10.1103/PhysRevE.63.042103). In that formalism the average free energy error would be $RT \cdot \ln(\phi_{\mathrm{eff}})$. Using this view ϕ_{eff} of 37%, 14% and 5% would correspond to errors of 1 $k_{\mathrm{B}}T$, 2 $k_{\mathrm{B}}T$, and 3 $k_{\mathrm{B}}T$, respectively. The details are, however, complicated by the fact that this “only” says something about how wrong the force field is, as viewed from the specific data, so the force field could still be wrong even if is ϕ_{eff} high. Nevertheless, it’s an important point that we now mention in the revised manuscript. (p. 22)

3) page 17: Here you use the same term "fit" with two different meanings:

"However, the fit to the SANS data at 0% D2O worsened slightly as the fit to SAXS data improved."

I would suggest to replace with first fit ("fit to the SANS") with "agreement with SANS data" or so, since you do not really "fit" but cross-validate against SANS. This would simplify the reading. Same for the phrase "For = 1.04, the fits to SANS datasets improved..."

Thank you very much for catching this. It was indeed not very clear and we have made the suggested changes. (p. 18)

4) page 18: "As discussed above, although the SAXS and SANS data are fully consistent..." Further up, you write that SAXS and SANS are rather mostly (and not fully) consistent. Revise?

We have modified the text on to be “overall consistent”. (p. 19)

5) page 20: "...a slightly higher value for the protein-water interaction strength best fitted data." -> fitted THE data?

Thanks for catching this; we have made this change. (p. 21)

Reviewer #2:

The authors report on a strategy combing coarse-grained MD using the Martini force field with SAXS/SANS data to study dynamic conformations of multidomain proteins. They demonstrate their approach with a well-studied three-domain protein TIA-1, for which high-resolution domain structures, SAXS and SANS data, including SANS data from segmentally deuterated protein, are available. The authors focus on an analysis of the dynamic ensemble of the three-domain protein in the absence of RNA, while a previous study has focused on the (more) rigid structural arrangements of the domains in the presence of RNA.

The authors performed coarse-grained MD keeping the domains semi-rigid and used a simplified back-mapping calculation to obtain an atomic description of structures to calculate Rg. The SAS and MD data were combined by Bayesian maximum entropy (BME) and quality of fits were assessed by a reduced chi_square.

The authors first optimize the regularization parameter theta, which scales the relative impact of data and simulation by reweighting in the BME approach. Thereby underestimation of calculated Rg compared to experimental data for the MD ensembles can be adjusted. Then they show that the value lambda for the protein-water interactions can be optimized in the Martini force-field to achieve best agreement with the experimental SAXS and SANS data. The authors are aware that the two adjustments for fitting experiment and simulation are not independent and may thus cause problems, but propose that the best agreement was obtained using this approach.

In a second part the author assess to role and information provided additional SANS data, when employing a MD/SAXS fitting protocol. They show that SANS can be used to aid and cross-validate the optimization of theta, while SAXS and SANS contributions are very similar. They show that the impact of contrast-matched SANS can be optimized and predicted, suggesting that in the specific example contrast-matched SANS data a sample with protonation of the second domain would provide additional information.

The manuscript is a carefully executed study combining state-of-the-art molecular dynamics simulation and BME with sparse experimental data for defining conformational ensembles of multidomain proteins. The computational procedures and analyses appear technically sound, although there are some questions (see below). The work focusses on the computational approach, while conclusions, interpretation and perhaps further validation of the structural ensembles is not attempted. Overall, the manuscript is interesting and should help to improve computational treatment of flexible multidomain proteins with SAXS/SANS data.

Thus, I recommend publication after the authors address the comments given below.

We thank the reviewer for his/her positive comments about our work, and for highlighting how we have attempted to provide a balanced view of the pros/cons of various approaches.

> *Specific comments:*
>
> *- For the reweighting by optimizing theta the authors state that simulation did not include the hydration shell, while this of course contributes significantly to the experimental SAXS data. This seems a gross inconsistency. Do the author imply that the reweighting protocol compensates for ignoring this somehow? Otherwise, it seems difficult to justify to optimize a parameter while ignoring hydration shell scattering. The authors should also perform the reweighting by considering the hydration shell to assess the effect of this in their approach.*

We thank the reviewer for this question, because it highlights that we were unclear in that part of our paper. To be clear, in all our comparisons to the SAXS and SANS data we do include solvent effects. For each structure we predict the scattering curves including solvent effects and average these before comparing to experiments. Thus, the agreement between calculated and experimental SAXS and SANS includes these (important) contributions.

However, for any ensemble we can also calculate the distribution of, for example, the radius of gyration of the protein coordinates. This calculation does not (per definition) take solvent into account. Thus, even for an ensemble that fits the data (taking solvent contributions into account), the average Rg calculated from the protein coordinates will not in general match the average Rg estimated e.g. from a Guinier analysis of the experimental data. Thus, we merely wanted to point out that fitting the (SAXS/SANS) data is more appropriate than fitting just the Rg.

We have changed the text to make this point clearer. (p. 10).

> *- The two fitting steps optimizing theta and lambda are not independent but rather inter-dependent as in both cases an optimal agreement between simulation and experiment is scored. How does this approach avoid a circular argument in that the two parameters are merely "fudging" in a not well-defined way? The authors seem to argue that a good force field does allow reweighting, where minimal reweighting may indicate the quality of the force field. Can this be used to generalize the approach and come up with a general recipe?*

This is a very good question to which we do not yet have a fully clear answer. They are indeed two different approaches, which may be combined. But the theoretical underpinnings are not fully clear yet. We have discussed this in a recent review (Ref 12; Orioli, Larsen et al, 2020, https://doi.org/10.1016/bs.pmbts.2019.12.006). On one side, tuning lambda potentially leads to a force field improvement that would transfer to other systems, but is limited by how flexible the fitting of the data can be. On the other hand, fitting the data using BME (via theta) is a fully system-specific correction. In the current paper we have taken the pragmatic approach and show that we get very similar results even if we chose a suboptimal lambda and then do more extensive reweighting with BME. But the exact details and formalism

of how to generalize these observations are still not fully clear. We now discuss this in the revised manuscript. (p. 22)

> *- The authors should describe which regions (residue numbers) were kept semi-rigid and which regions (linkers) were considered flexible. Is this justified, i.e. can the authors exclude that the linkers may not be completely flexible and, for example, exhibit some conformational features/propensities, or transiently interact with the domains?*

Regarding which residues are kept semi-rigid via the harmonic network and which are not, we now include this information in the revised manuscript (p. 4). We also note that the files for running simulations are available in the online repository that goes with the paper.

As for justifying exactly which residues to keep part of the folded domains we use several pieces of information. First, NMR data comparing the HSQC spectra of each RRM superposes perfectly with a spectrum of a construct with all three RRMs, demonstrating that they indeed behave as independent units (Wang et al, 2014; https://doi.org/10.1093/nar/gku193 and Freiburger et al, 2015; https://doi.org/10.1007/s10858-015-9981-0). Second, the structure of RRM2 is essentially the same whether in the complex of RNA (crystal structure, PDB ID 5O3J) or in the context of RRM2-RRM3 (NMR structure; PDB: 2MJN). Together, these observations suggest that it is a reasonable assumption to keep the structures of the domains relatively rigid and only allow for substantial flexibility in the individual domains. We now clarify these issues and arguments in the revised manuscript. (p. 9)

> *- How does the dynamic ensemble compare to other experimental data (if) available. Is there a way to validate the derived ensemble by experiments?*

We are not aware of any additional published data that we could compare to. Previous NMR experiments on the three-domain construct focused only on 15N HSQC experiments which do not provide much information because methods for calculating 15N and 1HN chemical shifts are rather inaccurate. We do, however, note the strong cross-validation between the SAXS and SANS data so that when we fit the SAXS data we independently refine our description of the SANS data. Further experiments on the relative orientation of the domains might require measurements of for example paramagnetic relaxation enhancement NMR and we mention this in the revised paper. (p. 23)

> *- In the introduction, the authors refer to a number of approaches to study dynamic protein systems of based on MD and/or SAXS/SANS data. Other groups have made relevant contributions, which should be listed as well, e.g. Delaforge E, et al. J Am Chem Soc 2015. PMID 26424125; Huang JR, et al. J Am Chem Soc 2014. PMID 24734879; Bertini I, et al (2010) J Am Chem Soc doi: 10.1021/ja1063923*

We thank the reviewer and now cite this work in our revised manuscript. (p. 2)
